# A Comparative Meta‐Analysis on the Association of lncRNAs MALAT1, HOTAIR, and AFAP1‐AS1 With the Risk of Developing Lymph Node Metastasis in Lung Cancer

**DOI:** 10.1002/cnr2.70091

**Published:** 2024-12-26

**Authors:** Anha Tasnim, Afra Anjum Sumaiya, Abdullah Al Noman, Anika Tahsin, Abdullah Al Saba, Rubaiat Ahmed, Tahirah Yasmin, A. H. M. Nurun Nabi

**Affiliations:** ^1^ Laboratory of Population Genetics, Department of Biochemistry and Molecular Biology University of Dhaka Dhaka Bangladesh

**Keywords:** AFAP1‐AS1, HOTAIR, lung cancer, lymph node metastasis, MALAT‐1

## Abstract

**Background:**

Numerous studies have demonstrated the significance of long noncoding RNA (lncRNA) in the development of cancer metastasis. The expression levels of many lncRNAs are elevated in metastatic lung cancer patients compared to non‐metastatic lung cancer patients.

**Objectives:**

The primary objective of the study was to investigate the association between the expression levels of three lncRNAs (MALAT1, HOTAIR, and AFAP1‐AS1) and lymph node metastasis (LNM) of lung cancer.

**Methods:**

Cell Press, PubMed, SpringerLink, Web of Science, and Google Scholar were explored to perform the literature search. After screening 1862 articles, 66 English‐language articles were selected based on the inclusion and exclusion criteria. From those articles, 17 publications comprising 1622 lung cancer patients were chosen for statistical analyses as well as quality assessment tests.

**Results:**

Forest plot analysis revealed that there was a significant difference in the incidence of LNM between the high and low MALAT1 expression groups (OR = 3.21, 95% CI: 1.34–7.67; random effects model). Significant differences were also observed in the incidence of LNM between patients with high and low HOTAIR expression levels (OR = 4.17, 95% CI: 1.47–11.82; random effects model). The expression level of AFAP1‐AS1 was found to be significantly associated with LNM in lung cancer (OR = 2.31, 95% CI: 1.39–3.85, random effects model). Additional analysis from GEPIA and GEO databases revealed that the expression levels of these lncRNAs vary according to the type of tumor tissue, organ of metastasis, and cancer stage. However, these databases show that the result for AFAP1‐AS1 is the most aligned with the meta‐analysis's findings. Furthermore, several quality assessment tests showed that the AFAP1‐AS1 studies are more reliable compared to the studies of other lncRNAs.

**Conclusion:**

This study suggested that LNM in lung cancer patients is associated mostly with an elevated AFAP1‐AS1 lncRNA level among the pool of three lncRNAs analyzed. Before these results can be implemented in a clinical setting, it is essential to conduct further validation and undertake comprehensive analysis to ensure robustness and reliability.

## 1 Introduction

Cancer is a leading cause of death worldwide, accounting for nearly 10 million deaths in 2020, or nearly one in six deaths [1]. Among different types of cancer, lung cancer is one of the most prevalent ones. It constitutes one of the most common cancers in Asia with a high mortality rate [2]. It is one of the most prevalent cancers in Bangladesh, ranked among the top two prevalent cancers in males in Bangladesh [3]. Several studies on various aspects of lung cancer have been conducted including its metastasis. Compared to people whose lymph nodes (LNs) are cancer‐free, patients with lymph node metastasis (LNM) from various malignancies have a greater probability of dying from cancer [4]. LN is marked as the most common site where lung cancer primarily metastasizes. Therefore, it is an important prognostic marker in lung cancer for metastasis and tumor staging [5]. The lack of adequate tumor biomarkers for early diagnosis and metastasis identification is still one of the most important challenges in lung cancer therapy [6]. Therefore, analyzing different risk factors for LNM of lung cancer has important therapeutic implications.

Recent studies have linked the role of long noncoding RNA (lncRNA) and the prognosis of various cancers, including lung cancer. Aberrant expression of some lncRNAs has been observed in metastatic lung cancer patients. Among them, only a handful have undergone functional characterization. They induce metastasis of several cancers by various molecular mechanisms both in vivo and in vitro [7]. LncRNAs are around 200 nucleotides in length and do not encode any protein [8]. These RNAs are broadly expressed and play important roles in gene regulation according to the evidence gathered during the previous 10 years. LncRNAs can modify chromatin function, control the assembly and operation of membrane‐less nuclear bodies, change the stability and translation of cytoplasmic mRNAs, disrupt signaling pathways, and more depending on their localization and the precise interactions with DNA, RNA, and proteins. Several of these processes eventually impact how genes are expressed in various biological and physio‐pathological situations, including neurological diseases, immunological responses, and cancer. LncRNAs can be targeted as potential biomarkers clinically because of their tissue and condition‐specific expression patterns [9, 10].

Several lncRNAs have been discovered that modulate the metastasis of lung cancer such as metastasis‐associated lung adenocarcinoma transcript‐1 (MALAT1), HOX antisense intergenic RNA (HOTAIR), colon cancer‐associated transcript 2 (CCAT2), BRAF activated noncoding RNA (BANCR), microvascular invasion in HCC (MVIH), SPRY4 intronic transcript 1 (SPRY4‐IT1), cancer‐associated region long noncoding RNA (CARLo‐5), plasmacytoma variant translocation 1 (PVT‐1), brain cytoplasmic RNA 1 (BCYRN1), and antisense noncoding RNA in the INK4 locus (ANRIL) [11, 12]. Recently, another lncRNA named actin filament‐associated protein 1 antisense RNA 1 (AFAP1‐AS1) has been identified, which has been associated with various cancers and can be used as a prognostic marker, including lung cancer. Its role has been found in epithelial to mesenchymal transition (EMT) and metastasis of lung cancer [13, 14].

Metastasis is one of the prominent causes of poor prognosis of many types of cancer. Researchers have been focusing on understanding the association between lncRNAs and metastasis of different cancers. Two crucial phenomena in cancer metastasis are EMT and local invasion of cancer cells. LncRNAs have been found to alter cell signaling and transcriptional pathways, causing unusual expression of transcription factors and regulatory proteins. Some lncRNAs such as MALAT1 and HOTAIR increase the efficiency of metastatic cancer cells to develop in distal sites. Moreover, some lncRNAs (XIST, BM, and MAYA) have been discovered to direct the specific distal organs where metastasis occurs, likely bones, LNs, central nervous system, and some more [15].

MALAT1 is one of the most extensively studied lncRNAs. It is associated with numerous diseases including cancer. A high level of MALAT1 expression is observed in cancers with high susceptibility to metastasize [16]. This RNA has an important role in metastasis of lung cancer by aiding invasion and EMT [17]. HOTAIR is a lncRNA that plays a role as an oncogenic molecule in different cancer cells, such as breast, gastric, colorectal, and cervical cancer cells [18]. The expression of HOTAIR regulates proliferation, survival, invasion, metastasis, and drug resistance in lung cancer cells. Increased expression of HOTAIR is found in tumor tissues of patients and is associated with LNM in patients with lung cancer [19]. AFAP1‐AS1 is a newly discovered lncRNA that contributes to the patho‐etiology of several cancers including esophageal adenocarcinoma, pancreatic carcinoma, and lung cancer. This lncRNA is found upregulated in metastasis and prognosis of cancer [20].

Previous research studies have suggested a role for these lncRNAs in lung cancer metastasis, but the evidence is not conclusive enough. This study aims to combine the understanding of the role of MALAT1, HOTAIR, and AFAP1‐AS1 lncRNAs in the metastasis of lung cancer by pulling the findings of the previous studies. Two of these lncRNAs (MALAT1 and HOTAIR) have been characterized over the years, whereas lncRNA AFAP1‐AS1 is a novel one extensively studied in Chinese populations [13]. Several meta‐analyses have been performed investigating the role of lncRNAs with various cancer types [21, 22] or with LNM [23]. However, as of today, there is no meta‐analysis focusing on the association of lncRNAs with the LNM of lung cancer specifically and comparing their effects. In this meta‐analysis, we have carried out a comparative analysis of the effect of these three lncRNAs on the LNM of lung cancer. This could provide insights into which lncRNA will be more appropriate to assess the risk for the metastasis of lung cancers among the pools of lncRNAs available. The population included in the meta‐analysis is all from Asia. Therefore, this analysis can also indicate the comparative prevalence of lncRNAs, which can be used as a selective potential biomarker for the diagnosis and treatment of metastatic lung cancer patients in the Asian population. To further validate the results, the Gene Expression Profiling Interactive Analysis (GEPIA) database was utilized to investigate the expression level of these lncRNA in normal tissue and lung tumor tissue. Additionally, the stage plot analysis of these lncRNA was conducted to assess the alteration in the expression levels as the cancer develops and progresses. Finally, survival plots were examined with an aim to predict the overall and disease‐free survival (DFS) of the patients. This brief bioinformatics analysis provided an overall insight into the role of these lncRNAs in various aspects of lung cancer development.

## 2 Methods

### 2.1 Literature Searching

A systemic search was conducted using the PRISMA guideline (Preferred Reporting Items for Systemic reviews and Meta‐Analyses) [24] across various electronic databases, namely, PubMed, SpringerLink, Web of Science, Google Scholar, and Cell Press. The keywords used in the search were “lncRNA”, “long noncoding RNA”, “long intergenic noncoding RNA”, “long intergenic RNA”, “lung cancer”, “lung cancer metastasis”, “cancer metastasis”, and “lymph node metastasis”. The names of the three lncRNAs “MALAT1”, “HOTAIR”, and “AFAP1‐AS1” as well as their full forms “metastasis associated lung adenocarcinoma transcript 1”, “HOX antisense intergenic RNA”, and “actin filament‐associated protein antisense RNA1”, respectively, were searched individually and with other keywords. Alternative spelling (i.e., MALAT1 or MALAT‐1) was considered by searching with the “OR” operator. The “AND” operator was used to increase the specificity of the search results so that only LNM of lung cancer is included in the result. Two researchers independently collected the literature by following the PRISMA protocol, and then their findings were combined. A senior researcher was consulted to resolve any disagreements and reach a consensus. He assessed the article by reading the title, abstract, tables, and figures based on inclusion and exclusion criteria to decide whether a study should be selected or discarded. The search was completed on June 9th, 2024, and no lower date limit was used.

### 2.2 Selection Criteria

For primary selection, certain exclusion and inclusion criteria were adopted. Articles with duplication, review papers, and irrelevant papers were excluded. The investigators initially looked at the titles and abstracts while screening the articles. Any irrelevant articles were excluded. Then, the full‐text papers with special emphasis on the methodologies and result section were read to assess if they met the inclusion and exclusion criteria. Some literature studies were collected by screening reference articles of review papers on the corresponding topic. One of the ways to assess literature credibility was by analyzing Q factors of the journals. Literature studies included for analysis had been published from journals having Q factors of Q1 to Q3 up to June 2024. They were primary journal articles all in English and peer‐reviewed.

Finally, articles fulfilling the following criteria were selected: (i) articles contained sufficient data for computing odds ratios (ORs) and corresponding 95% confidence intervals (CIs), (ii) the data on any of the three lncRNAs’ expression levels were available, (iii) the technique of obtaining expression data in patients was either qRT‐PCR or ISH (in situ hybridization) technique, (iv) information on the normalization of lncRNA expression was present, (v) metastatic patients were categorized into high and low groups according to the expression data, (vi) samples obtained from cancer patients were either tissue or blood, and (vii) clinicopathologic parameters were available.

Exclusion criteria were as follows: (i) letters, expert opinions, and case reports, (ii) studies investigating only the molecular structure and functions of lncRNAs, (iii) studies without data providing high–low grouping of patients based on expression, (iv) duplicates or continued work of previous publications, and (v) metastasis indicated by cellular features in in vitro culture.

### 2.3 Retrieval of the Data

Data regarding solely LNM was retrieved due to the importance and severity of LNM in lung cancer. The following information was collected from each eligible study: author, publication year, country of origin, cancer type, sample size (total number of patients), number of patients and metastatic patients with high lncRNA expression, number of patients and metastatic patients with low lncRNA expression, p‐value, organ of metastasis, and detection method.

### 2.4 Statistical Analysis and Quality Assessment

A forest plot was generated to show the OR of the individual study and the overall pooled estimate. The pooled OR was used to test the association between the expression level of lncRNA and the probability of developing LNM from lung cancer. The null hypothesis of this test was that there is no association between the expression level and probability of developing LNM from lung cancer (OR = 1). The statistical significance of the OR was defined as the CI value being more than 1. The forest plot also detected the heterogeneity of the data, evaluated by the chi‐square Q test (χ^2^) and I^2^ statistic. For the χ^2^ test, a p‐value of less than 0.05 indicated significant heterogeneity. When there was high heterogeneity, indicated by an I^2^ value of greater than 50%, a random effects model was taken into consideration for the outcome. Otherwise, a common effect model was considered. The extent of heterogeneity was visualized using the Galbraith radial plot. It identified which study acted as an outlier and had the greatest impact on the heterogeneity of the outcome.

The quality of the selected publications was assessed by the NOS (Newcastle‐Ottawa Quality Assessment Scale) score [25]. It considered the information about the selection method, comparability of studies, and outcome. Specific stars were allocated for different questions. The standard of points considered here: 8 to 9 points for low risk of bias, 6 to 7 points for medium risk of bias, and 5 or less points for high risk of bias. Any discrepancy in the scoring was discussed and resolved by reaching a consensus. The questionnaire for assessing the NOS score is mentioned in Supplementary file Data S1.

To assess the publication bias for studies of all three lncRNAs, a funnel plot was generated. Begg's test [26] and Egger's test [27] were carried out to determine the asymmetry of the funnel plot and assess the extent of publication bias by using the rank correlation test and linear regression test, respectively. A p‐value of less than 0.05 was considered to be statistically significant and indicative of the presence of publication bias. Furthermore, to evaluate the consistency of the overall result of the meta‐analysis, a sensitivity analysis plot was generated by systemically excluding one study at a time and observing the effect on the pooled estimate. The overall meta‐analysis was considered to be robust and not sensitive to a specific study if the pooled estimate remained relatively consistent.

All the statistical analysis as well as the quality assessment tests were done by R programming using libraries such as, metafor, meta, tidyverse, ggplot2, etc.

### 2.5 Validation of Results by GEPIA and Gene Expression Omnibus (GEO) Databases

In order to validate the outcome further, the expression profiles of these lncRNAs in normal and lung cancer tissue were retrieved from GEPIA (http://gepia.cancer‐pku.cn/). It is an interactive web server for analyzing the RNA sequencing expression data of The Cancer Genome Atlas (TCGA) and the GTEx projects, using a standard processing pipeline [28]. Pathological stage plots were deduced as violin plots based on the patient pathological stage. The method for differential gene expression analysis was one‐way ANOVA, using the pathological stage as a variable for calculating differential expression. Survival plots denoting the influence of lncRNA expression on overall survival (OS) and DFS in lung cancer patients were generated as Kaplan–Meier (K–M) curves. The Log‐rank test or the Mantel–Cox test was used to test the hypothesis that there is association between lncRNA expression and the survival of lung cancer patients, with the null hypothesis being that there is no association between lncRNA expression and the survival of lung cancer patients. For the analyses, the median value of lncRNA expression was set as a cutoff and *p*‐value < 0.05 for the hazard ratio was considered statistically significant. Both lung adenocarcinoma (LUAD) and lung squamous cell carcinoma (LUSC) datasets of TCGA were selected for analyses as lung cancer. Log_2_ (TPM + 1) or log_2_ of transcript per million was used for the log scale.

Expression data for comparison between the expression of lung cancer tissue and normal tissue were also extracted from the GEO database and analyzed with GEO2R. It is an online interactive tool that compares two or more groups of samples within a GEO series to find genes that are differentially expressed under different conditions. GEO2R, utilizing the limma package, employs a moderated t‐statistic to assess differential gene expression, generating p‐values that are then adjusted using the Benjamini–Hochberg (FDR) method. The change in the expression level of three lncRNAs was presented as logarithmic fold change or log_2_ (FC) in a bar plot. A positive value of log_2_ (FC) indicates an increase in the expression level, whereas a negative value denotes a decrease. The null hypothesis of this test was that there is no difference in gene expression levels between lung cancer tissue and normal tissue, whereas the alternative hypothesis was that there is a difference in gene expression levels between lung cancer and normal tissue. A two‐sided test was performed to identify genes that were either upregulated or downregulated in lung cancer tissue compared to normal tissue. The statistical significance of the change was considered as p‐value < 0.05.

## 3 Results

### 3.1 Study Selection

**FIGURE 1:**
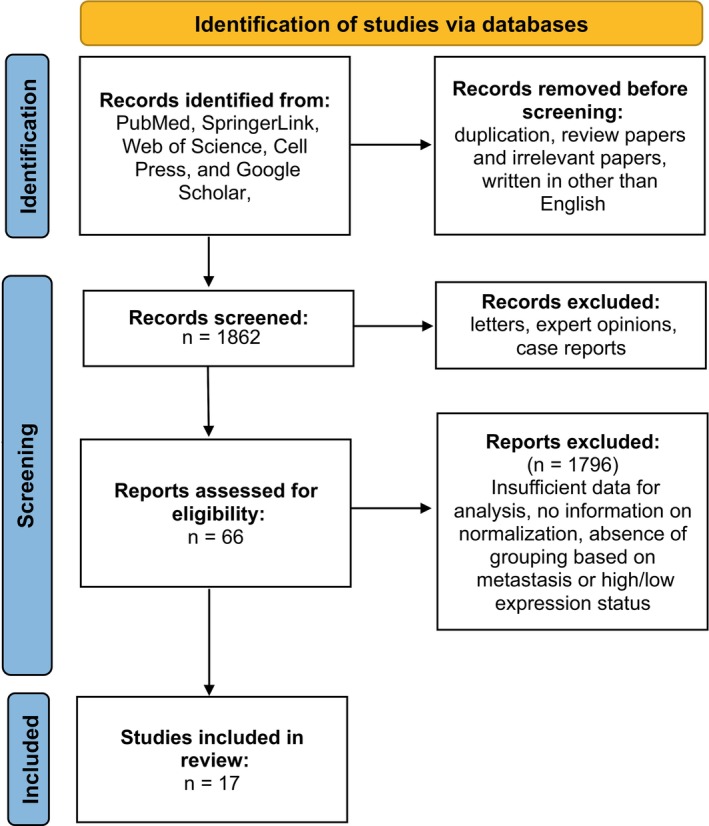
Flow diagram of selecting eligible articles according to the PRISMA protocol.

Database searches resulted in a total of 1862 articles. In the search results for MALAT1, HOTAIR, and AFAP1‐AS1 lncRNAs, there were 1111, 576, and 175 publications, respectively. The titles and abstracts were investigated. After excluding letters, expert opinions, case reports, and other irrelevant studies, 66 studies were chosen for the full‐text review. By examining the introduction, methodologies, and result section, 17 out of these 66 studies fulfilled the inclusion criteria and made it through the final screening. Among them, six studies were for MALAT1 [29, 30, 31, 32, 33, 34]; four for HOTAIR [35, 36, 37, 38]; and seven for AFAP1‐AS1 [39, 40, 41, 42, 43, 44, 45]. The flow diagram of the selection process according to the PRISMA protocol is represented in Figure 1.

### 3.2 Characteristics of Included Studies

For dividing the metastatic patients into two groups, i.e., with high and low lncRNA expression, several methods were adopted in the included articles: (i) high expression and low expression, according to a HOTAIR/ACTB ratio of 1.368 in tumor tissues, obtained by the ROC method; (ii) high expression group: expression ratio ≥ median ratio; and low expression group: expression ratio≤median ratio; and (iii) high expression levels in their tumor tissues were equal to or more than 2‐fold that of corresponding normal tissues.

In most studies, RT‐PCR was adopted as the assay technique and the sample was taken from cancer tissue or blood. In some of the articles, the in situ hybridization technique was used to measure the expression level. Moreover, the lncRNA expression level was normalized by GAPDH, U6, and ACTH level.

### 3.3 General Characteristics of the Patients

A total of 1622 patients were included in the present study for three lncRNAs of interest (644, 227, and 751 patients for MALAT1, HOTAIR, and AFAP1‐AS1, respectively) (Table 1). In the case of MALAT1, 46.85% of patients with high expression levels developed metastasis, whereas 40% of the low expression group had metastasis. 53.85% of patients with high HOTAIR expression had metastasis but the number was only 23.53% for the low expression group. Finally, for AFAP1‐AS1, 63.21% and 43.64% of patients with high and low expression levels, respectively, developed metastasis. These values indicated that patients with high expression levels possess more tendency to develop metastasis compared to patients with low expression levels. This indication was further assessed by statistical analysis.

**TABLE 1:** Summary of the characteristics of the articles included in this study.

lncRNA	Author	Year	Country	Cancer type	Total patients	Patients with high expression		Patients with low expression		*p* value	Detection method
						Total	With LNM	Total	With LNM		
**MALAT1**	Jen et al.	2017	Taiwan	Lung Cancer	124	110	48	14	2	0.029	qRT‐PCR
	Yang et al.	2019	China	NSCLC	326	232	84	94	44	0.076	qRT‐PCR
	Chen et al.	2017	China	Lung cancer	42	21	14	21	5	0.005	qRT‐PCR
	Xiao et al.	2019	China	LUAD	39	20	16	19	8	0.015	qRT‐PCR
	Tang et al.	2018	China	NSCLC	36	14	11	22	9	0.0407	qRT‐PCR
	Zhang et al.	2017	China	NSCLC	77	47	35	30	12	0.002	qRT‐PCR
**HOTAIR**	Ono et al.	2014	Japan	SCLC	35	12	10	23	10	0.03	qRT‐PCR
	Chen et al.	2021	China	NSCLC	32	21	19	11	5	0.008	qRT‐PCR
	Lizuka et al.	2022	Japan	LUAD	83	41	13	42	11	0.56	qRT‐PCR
	Nakagawa et al.	2013	Japan	NSCLC	77	17	7	60	6	0.047	qRT‐PCR
**AFAP1‐AS1**	Zhong et al.	2021	China	LUSC and LUAD	174	72	48	102	60	0.0248	ISH, qRT‐PCR
	Yin et al.	2018	China	NSCLC	92	46	27	46	24	0.529	qRT‐PCR
	Peng et al.	2017	China	Lung cancer	98	79	52	19	7	0.022	qRT‐PCR
	Deng et al.	2015	China	NSCLC	121	66	37	55	17	0.006	qRT‐PCR
	Yu et al.	2019	China	NSCLC	96	48	29	48	18	0.025	qRT‐PCR
	Li et al.	2017	China	NSCLC	126	66	48	60	16	0	RT‐PCR
	Huang et al.	2019	China	NSCLC	44	28	15	16	9	0.041	RT‐PCR

Abbreviations: ISH = in situ hybridization; LUAD = lung adenocarcinoma; LNM = lymph node metastasis; LUSC = lung squamous cell carcinoma; SCLC = Small cell lung cancer; NSCLC = non‐small cell lung cancer.

**FIGURE 2:**
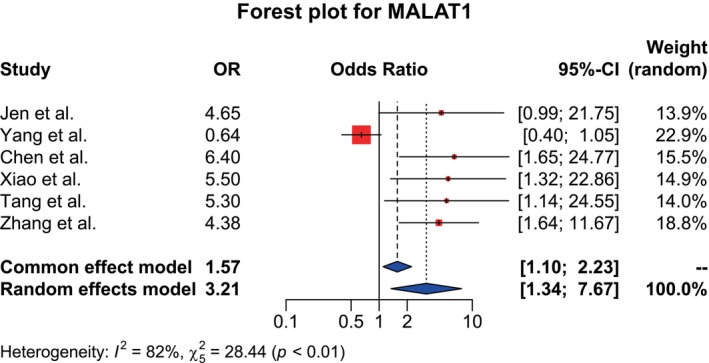
Forest plot showing the association between the MALAT1 expression level and LNM. High heterogeneity (I^2^ = 82%, χ^2^ = 28.44 (*p* < 0.01)) denotes a random effects model. Pooled OR = 3.21, 95% CI: (1.34–7.67), indicating a statistically strong association between them.

**FIGURE 3:**
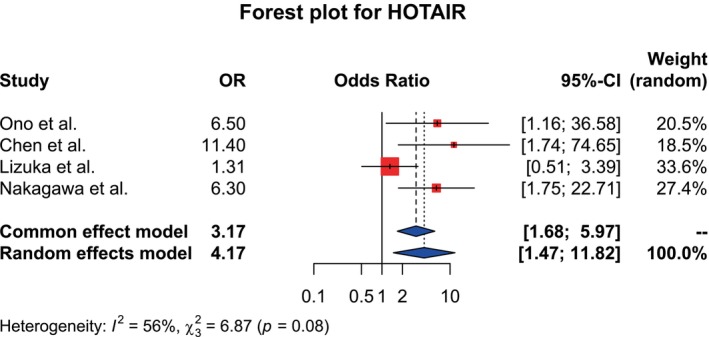
Forest plot showing the association between the HOTAIR expression level and LNM. I^2^ = 56% indicates high heterogeneity, so the random effects model was chosen. However, the χ^2^ test did not reveal any significant heterogeneity (χ^2^ = 6.87, *p* = 0.08). Pooled OR = 4.17, 95% CI: (1.47–11.82), indicating a significant association between them.

**FIGURE 4:**
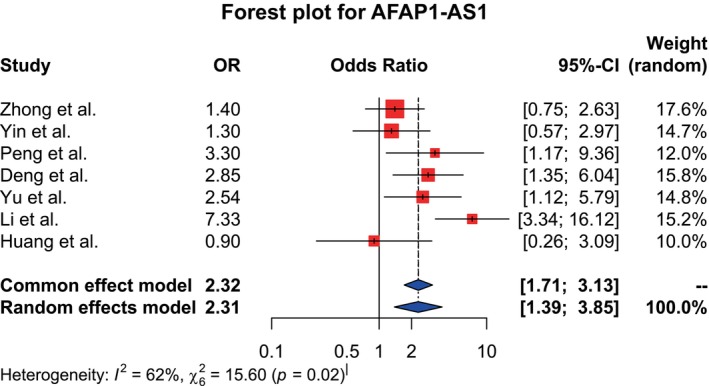
Forest plot showing the association between the AFAP1‐AS1 expression level and LNM. The random effects model was implicated as I^2^ = 62%, χ^2^ = 15.60 (*p* = 0.02), indicating high heterogeneity. Pooled OR = 2.31, 95% CI: (1.39–3.85), indicating a statistically significant association between them.

Forest plots were generated for all three lncRNAs (Figures 2, 3, 4 for datasets of MALAT1, HOTAIR, and AFAP1‐AS1, respectively).

### 3.4 Association Between MALAT1 Expression and LNM Development

For MALAT1, a total of six studies were included. As a result of the high heterogeneity finding (I^2^ = 82%; χ^2^ = 28.44, *p* < 0.01) of the analysis, the random effects model was chosen. The pooled OR was determined by the model to be 3.21, with a 95% CI of (1.34–7.67). It suggested that the high expression group had statistically significant susceptibility to develop LNM compared to the low expression group.

### 3.5 Association Between HOTAIR Expression and LNM Development

Statistical analysis for HOTAIR was done by including four studies. Although the χ^2^ test did not reveal any significant heterogeneity (χ^2^ = 6.87, *p* = 0.08), the I^2^ test revealed high heterogeneity (I^2^ = 56%). Therefore, the random effects model was adopted. According to the model, the pooled OR was found to be 4.17 with a 95% CI (1.47–11.82). According to this, patients with high HOTAIR expression had a significantly higher tendency to develop LNM compared to patients with low HOTAIR expression levels.

### 3.6 Association Between AFAP1‐AS1 Expression and LNM Development

Statistical analysis for AFAP1‐AS1 was also done by taking seven studies. The analysis generated high heterogeneity (I^2^ = 62%; χ^2^ = 15.60, *p* = 0.02), so the random effects model was adopted. According to the model, the pooled OR was found to be 2.31 with a 95% CI (1.39–3.85), suggesting that there is a statistically significant association between the high AFAP1‐AS1 expression level and the tendency to develop LNM.

### 3.7 Heterogeneity Analysis

**FIGURE 5:**
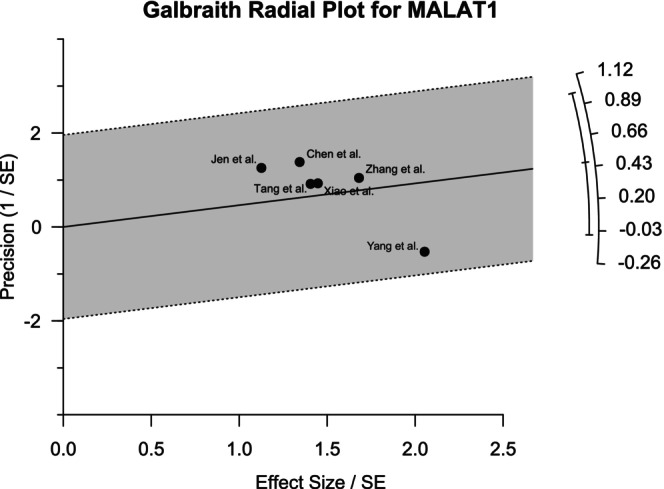
Galbraith plot for the six studies of MALAT1 revealing the heterogeneity among them. The outlier The study of Yang et al was observed to be the outlier.

**FIGURE 6:**
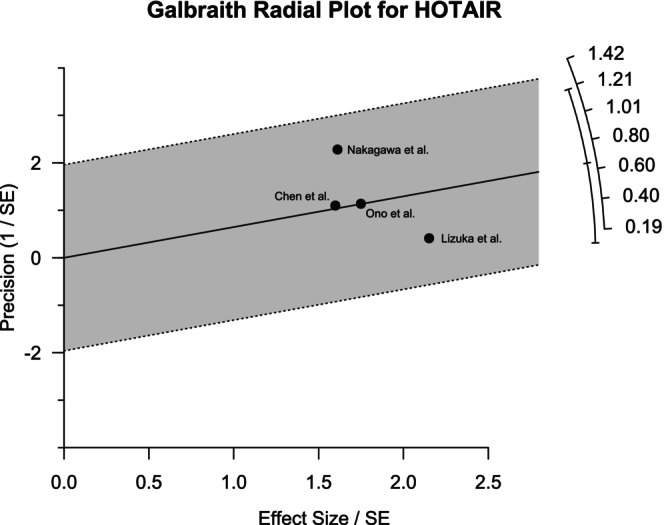
Galbraith plot for the four studies of HOTAIR revealing the heterogeneity among them. Two studies, Nakagawa et al. and Lizuka et al., were observed to be mostly responsible for heterogeneity.

**FIGURE 7:**
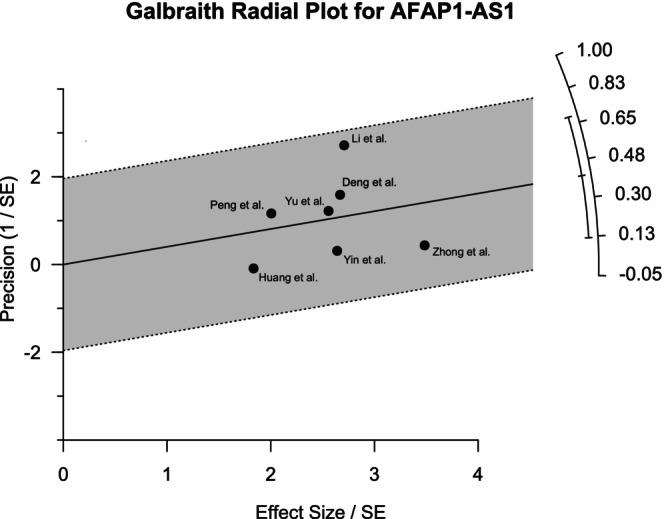
Galbraith plot for the seven studies of AFAP1‐AS1 revealing the heterogeneity among them. The study by Li et al. was found to be contributed most for heterogeneity.

Galbraith plots were generated to visualize the heterogeneity among different studies. For MALAT1, the outlier was observed to be the study of Yang et al. (Figure 5). The forest plot generated by excluding this study showed 0% heterogeneity with a pooled OR of 5.05 (95% CI: 2.80–9.10) for the common effect model, which validates the finding of the Galbraith plot. Figure 6 shows that both the studies of Nakagawa et al. and Lizuka et al. contributed to the heterogeneity. Regarding the AFAP1‐AS1 studies, the study by Li et al. contributed most to the heterogeneity, as revealed in Figure 7. However, the effects of the outliers for these two lncRNAs were not as visually prominent as that for MALAT1.

### 3.8 Assessment of Publication Bias

**FIGURE 8:**
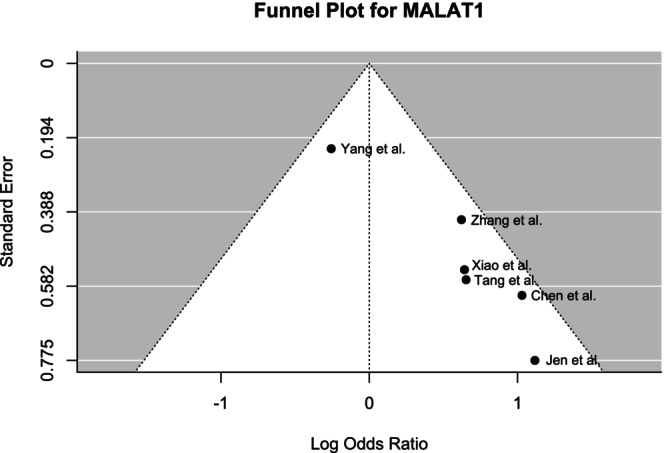
Funnel plot showing the publication bias of included studies for MALAT1 demonstrating asymmetry.

**FIGURE 9:**
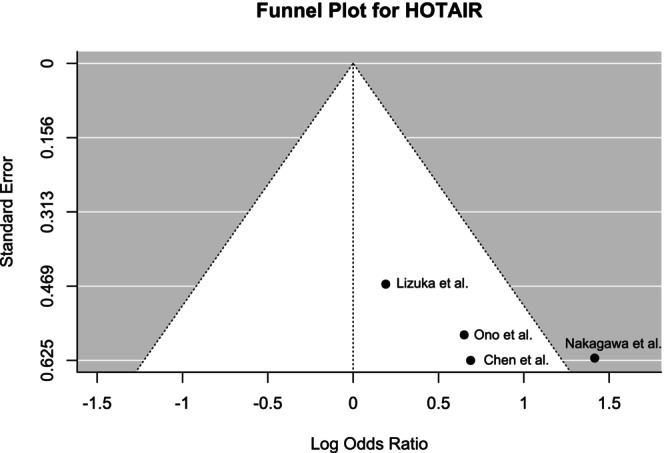
Funnel plot showing the publication bias of included studies for HOTAIR demonstrating overall symmetry.

**FIGURE 10:**
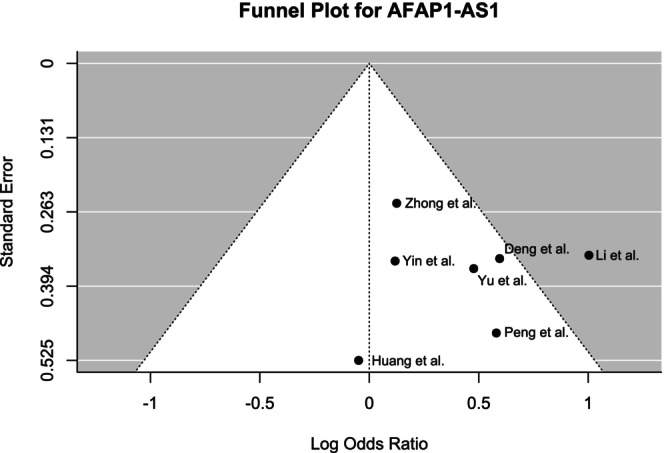
Funnel plot showing the publication bias of included studies for AFAP1‐AS1 demonstrating overall symmetry.

Funnel plots were generated to determine the publication bias (Figures 8, 9, 10 for MALAT1, HOTAIR, and AFAP1‐AS1, respectively). Although Begg's test did not reveal any significant asymmetry for MALAT1 (*p* = 0.35), the asymmetry was found to be statistically significant by Egger's test (*p* < 0.01). The funnel plot for HOTAIR did not show any statistically significant asymmetry according to both Begg's test (*p* = 0.17) and Egger's test (*p* = 0.09). Similarly, no significant asymmetry was found by Begg's test (*p* = 0.88) and Egger's test (*p* = 0.95) for the funnel plot of AFAP1‐AS1.

### 3.9 Data Quality Assessment

The NOS score was evaluated to assess the data quality. Out of the 17 studies, 11 had a score of 8 on a scale of highest 9 points, indicating that they have a low risk of bias. Six studies scored 7 denoting a medium risk of bias. Among these six studies, three were from MALAT papers, one from HOTAIR, and two from AFAP1‐AS1. Scores for different criteria such as selection, comparability, and outcome along with the total score for individual study are presented in Table 2.

**TABLE 2:** NOS score of 17 studies for data quality assessment along with points obtained by them in selection, comparability, and outcome criteria.

Question and marks		Selection				Comparability	Outcome			Total score
lncRNA	Author	1	2	3	4	1	1	2	3	
MALAT1	Zhang et al.	*	*	*	*	**	*	*		8
	Tang et al.	*	*	*	*	*	*	*		7
	Jen et al.	*	*	*	*	**	*	*		8
	Yang et al.	*	*	*	*	**	*	*		8
	Chen et al.	*	*	*	*	*	*	*		7
	Xiao et al.	*	*	*	*	*	*	*		7
HOTAIR	Ono et al.	*	*	*	*	**	*	*		8
	Nakagawa et al.	*	*	*	*	**	*	*		8
	Chen et al.	*	*	*	*	*	*	*		7
	Lizuka et al.	*	*	*	*	**	*	*		8
AFAP1‐AS1	Huang et al.	*	*	*	*	**	*	*		8
	Li et al.	*	*	*	*	*	*	*		7
	Yu et al.	*	*	*	*	**	*	*		8
	Zhong et al.	*	*	*	*	*	*	*		7
	Yin et al.	*	*	*	*	**	*	*		8
	Peng et al.	*	*	*	*	**	*	*		8
	Deng et al.	*	*	*	*	**	*	*		8

*Note:* The asterisks denote the scores assigned to each question according to the questionnaire (a maximum of one asterisk for each question under selection and outcome criteria and a maximum of two asterisks for the question under comparability criteria). The use of color is for visualization purposes and does not carry any analytical significance.

### 3.10 Sensitivity Analysis

**FIGURE 11:**
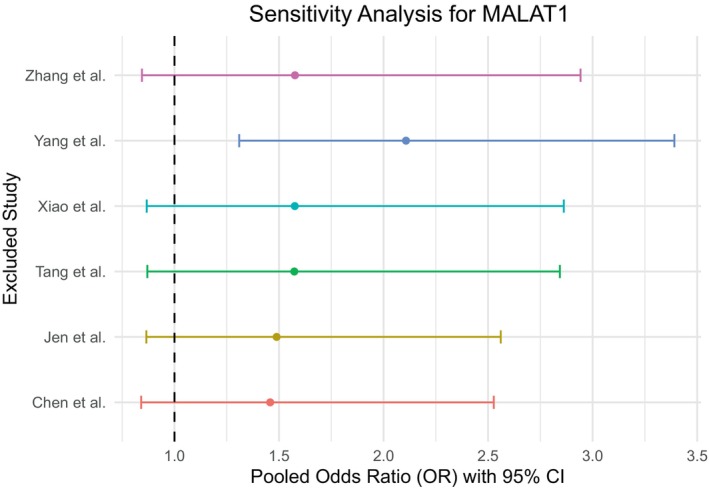
Sensitivity analysis for the studies of MALAT1 showed the pooled estimate was sensitive toward the study of Yang et al.

**FIGURE 12:**
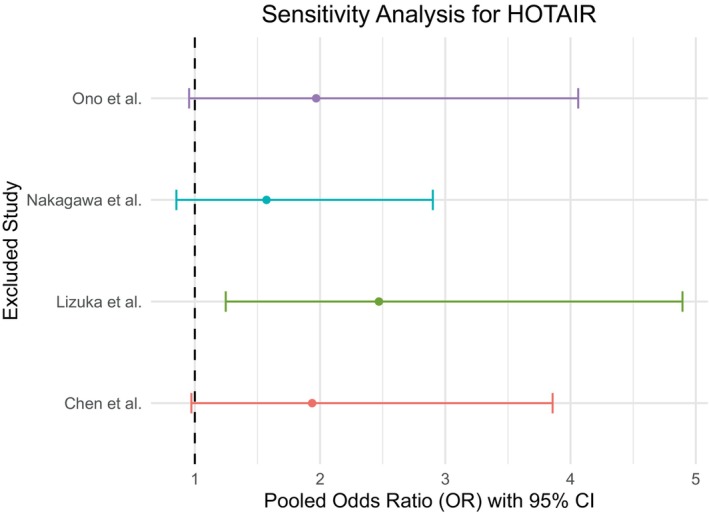
Sensitivity analysis for the studies of HOTAIR revealed no substantial effect of any single study on the pooled odds ratio.

**FIGURE 13:**
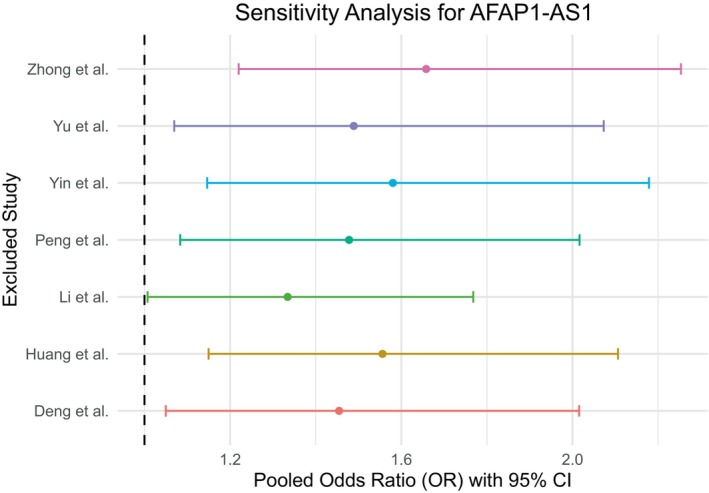
Sensitivity analysis for the studies of AFAP1‐AS1 showed no significant effect of any single study on the pooled odds ratio.

Sensitivity analysis for MALAT1 studies (Figure 11) revealed that the pooled estimate is sensitive toward one study (Yang et al.) as the pooled OR excluding this study was visually different from the pooled estimates excluding any other study. On the other hand, the analysis showed no substantial effect of a particular study on the pooled estimate in the case of both HOTAIR studies (Figure 12) and AFAP1‐AS1 studies (Figure 13).

### 3.11 Results of the Analysis From the GEPIA and GEO Databases

**FIGURE 14:**
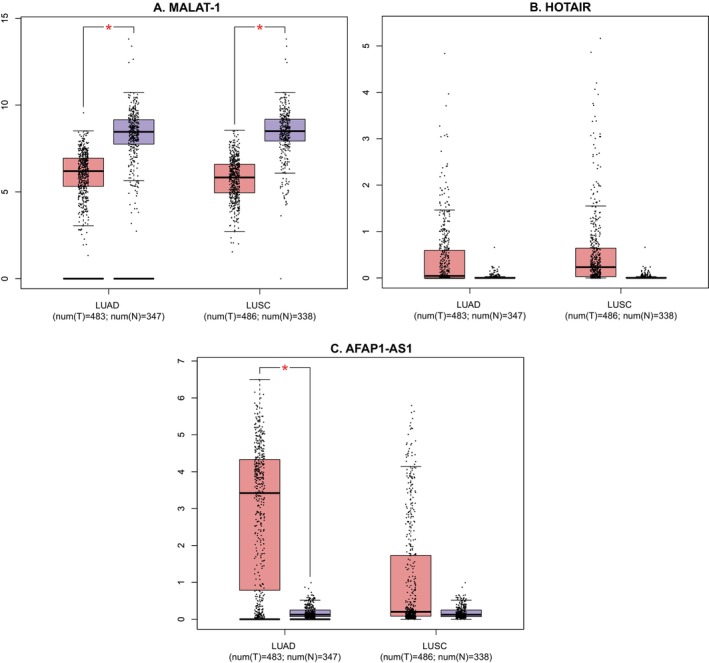
Expression of three lncRNAs in two types of cancer tissue (LUSC = lung squamous cell carcinoma, and LUAD = lung adenocarcinoma) compared to normal tissue. Statistical significance (*p* < 0.05) was denoted by an asterisk (*). A. The expression of MALAT1 was significantly lower in LUAD and LUSC than in normal tissue. B. The expression of HOTAIR was increased in both cancer tissue types compared to normal tissue, but the difference was not statistically significant. C. The expression of AFAP1‐AS1 was higher in both LUAD and LUSC tissue when compared to normal tissue, but statistical significance was found in the case of LUAD only.

The expression levels of three lncRNAs in various types of lung tumor tissues were compared to those of normal tissues using the GEPIA database. The expression profile for both HOTAIR and AFAP1‐AS1 revealed that their expression was higher in tumor tissue compared to normal tissue, although only AFAP1‐AS1 in LUSD had a statistically significant difference. However, MALAT1 in both lung cancer tissue types was significantly lower than in normal tissue (Figure 14).

**FIGURE 15:**
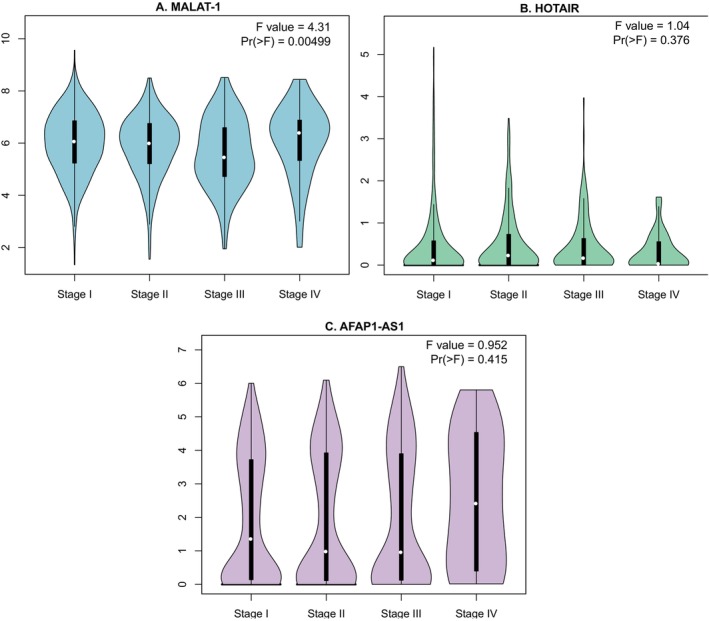
The pathological stage plot illustrating lncRNA expression at various stages of the development of cancer. Pr( < F) represents the statistical significance of F‐statistic. Pr( < F) values of less than 0.05 indicate a statistically significant difference in the lncRNA expression levels across the different pathological stages. The four stages of cancer development showed significant differences in MALAT1 expression (*p* < 0.01), with stage III showing the lowest expression (A). The expression of HOTAIR (B) did not differ significantly across the cancer stages. The expression of AFAP1‐AS1 (C) was elevated in stage IV of cancer; however, the change was not statistically significant.

The pathological stage plot in Figure 15 showed that MALAT1 expression differed significantly throughout the four stages of cancer development (*p* = 0.005), with stage III having the lowest expression. However, the differences in the expression level of HOTAIR throughout the four stages of cancer development was not statistically significant (*p* = 0.38). The expression level of AFAP1‐AS1 was elevated in the last stage of cancer, although the change was not found to be statistically significant. The stage plot showed no visible difference in the expression level of HOTAIR throughout the cancer stages.

**FIGURE 16:**
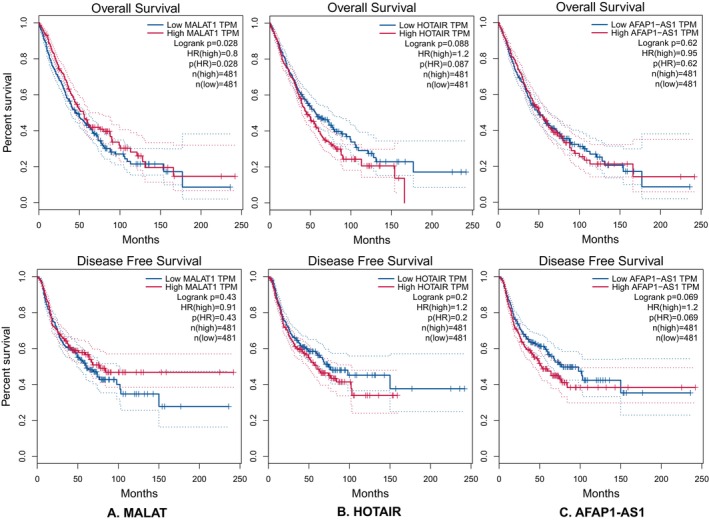
The Kaplan–Meier (K–M) curves for overall survival (OS) and disease‐free survival (DFS) illustrating the association of the lncRNA expression level with risk of cancer development. HR(high) indicated the hazard ratio; HR(high) < 1 denoted that high expression is linked with the probability of better survival, whereas HR(high) > 1 represented the higher probability of death. Low MALAT1 expression was found to be linked to lesser survival probability of cancer patients [HR(high) < 1], with statistical significance observed only for OS (p < 0.05). Conversely, there was no association between HOTAIR expression and OS and DFS (*p* > 0.05). Similarly, no association was observed between AFAP1‐AS1 expression and OS and DFS of patients (p > 0.05).

The K–M curves in Figure 16 represent the association between lncRNA expression and the OS and DFS of cancer patients. From the figure, it is revealed that the high expression of MALAT1 [HR(high) < 1] is associated with better survival in cancer patients. However, statistical significance was only found in the case of OS (*p* < 0.05). In contrast, there was no association between HOTAIR expression and OS and DFS of patients (*p* > 0.05 for HR (high) of both OS and DFS). There was also no association between AFAP1‐AS expression and OS and DFS cases, as the HR for both OS and DFS cases were not statistically significant.

**FIGURE 17:**
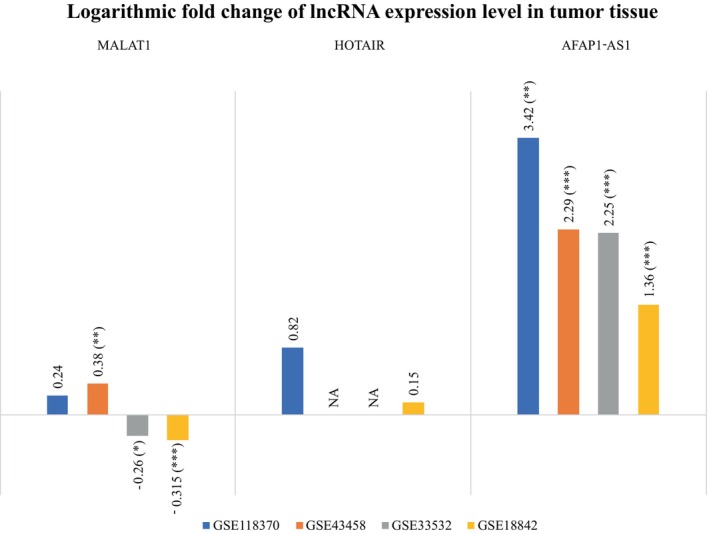
Bar plot showing the log(FC) or logarithmic fold change in the expression level of three lncRNAs in tumor tissue compared to normal tissue. The length of the bar represents the extent of log(FC); the values were written as well. The statistical significance was denoted by asterisks [p < 0.05 denoted by (*), *p* < 0.01 denoted by (**), and *p* < 0.001 denoted by (***)]. Two datasets revealed an increase in MALAT1 in tumor tissue; however, only one of them was statistically significant (*p* < 0.01 for GSE43458). In contrast, the other two datasets indicated a decline in their expression levels (*p* = 0.03 and *p* < 0.001 for GSE33532 and GSE18842, respectively). The logarithm of the fold change data from HOTAIR was available for just two datasets. However, there was no statistically significant change. All four datasets revealed that the expression level of AFAP1‐AS1 was significantly higher in tumor tissue than in normal tissue (*p* = 0.001 for GSE118370 and *p* < 0.001 for the other three).

The results obtained from the GEO database are presented in Figure 17. Four different datasets (GSE118370, GSE43458, GSE33532, and GSE18842) were found to contain the data of expression levels of three lncRNAs in tumor tissue in comparison to normal tissue. These four datasets contained data from a total of 101 normal tissues and 151 lung tumor tissues. MALAT1 was found to be increased in tumor tissue according to two datasets but the value of fold change was statistically significant in only one of them (*p* < 0.01 for GSE43458). On the contrary, its expression level was found to be decreased according to the other two datasets (*p* = 0.03 and p < 0.01 for GSE33532 and GSE18842, respectively). The logarithm of fold change data of HOTAIR was available for only two datasets. However, no statistically significant change was observed. The expression level of AFAP1‐AS1 was found to be significantly increased in tumor tissue compared to normal tissue according to all four datasets (*p* = 0.001 for GSE118370 and *p* < 0.001 for the other three).

## 4 Discussion

Numerous earlier research studies have shown the function of various lncRNAs in cancer growth, invasion, and metastasis, illuminating their mode of action.

MALAT1 has been shown to bind polycomb repressive complex 2 (PRC2) components, which catalyze histone H3K27 methylation, and it plays an important role in transcriptional repression and cancer. Moreover, it has been found to regulate EMT, migration, invasion, and metastasis of cancer cells by modulating Wnt signaling. A number of studies reported that MALAT1 functions through sponging miRNAs, including miR‐145, miR‐1, miR‐202, miR‐200c, miR‐206, miR‐204, and others [46]. HOTAIR promotes matrix metalloproteinase (MMP‐2 and MMP‐9) activity for cell motility and the capacity to dissolve the basement membrane contributing to the invasion of cancer cells [47]. HOTAIR along with miR‐196a promotes the dedifferentiation of lung epithelial cells during the development of lung tumors. It supports cell growth and survival in LUAD cells by suppressing p21WAF1/CIP1, which is a modulator of p53‐induced growth arrest and death in response to DNA damage [48].

A number of studies demonstrated that AFAP1‐AS1 was linked to a poor prognosis and promoted cell invasion and metastasis in lung cancer by controlling the integrity of actin filaments. Additionally, in LUAD, a subtype of non‐small cell lung cancer, knocking down AFAP1‐AS1 decreased the tumor cells’ ability to invade and migrate as well as promoted tumor cell apoptosis by altering the expression of several small GTPase family members and molecules in the actin cytokeratin signaling pathway [49].

Subsequently, this meta‐analysis aimed to investigate the role of these three lncRNAs in developing LNM from lung cancer. Total 1622 patients were included in the study for three different lncRNAs considering their expression level and metastatic status of the patients. Statistical analysis with R revealed that high expression of all three lncRNAs—MALAT1, HOTAIR, and AFAP1‐AS1—significantly affects the development of metastasis (pooled OR = 3.21, 4.17, and 2.31, respectively, with a 95% CI of (1.34–7.67), (1.47–11.82), and (1.39–3.85), respectively).

Heterogeneity analysis by the Galbraith plot demonstrated the outliers that contributed most to the heterogeneity among the studies. Galbraith plots revealed one outlier each for MALAT1 and AFAP1‐AS1 and two for HOTAIR studies. These outliers had highly different ORs compared to others. Moreover, the statistical significance of the correlation between lncRNA expression and LNM of the patients among the selected papers varied widely. This might be a contributing factor to the heterogeneity. Sensitivity analysis revealed that the results were not sensitive to any specific paper except one study for MALAT1. The data quality of the papers used in this study was very good as assessed by NOS scoring. The funnel plot generated by R programming showed overall symmetry for HOTAIR and AFAP1‐AS1 and asymmetry for MALAT1. The plots were further validated by Egger's test and Begg's test. The test result for HOTAIR and AFAP1‐AS1 provided a satisfactory outcome according to both Egger's and Begg's tests. However, for MALAT1 studies, the asymmetry was considerable according to Egger's test.

In an attempt to corroborate the results, the lncRNA expression patterns in various tissue types (cancerous vs. non‐cancerous tissue) and during the various phases of cancer development were taken from the GEPIA database. The results of this meta‐analysis appeared to contradict the finding that MALAT1 expression was lower in LUAD and LUSC tissue as compared to normal tissue. However, it can be explained by the fact that the GEPIA database only displayed a comparison between tumor and normal tissue, not a distinction between metastatic and non‐metastatic tissue. Previous research revealed that the expression of MALAT1 can also vary depending on the site of metastasis, as evidenced by the reduced expression of the gene found in the tissues of metastatic LNs compared to bone and brain metastasis [50]. Additionally, as the stage plot illustrated, the expression level was also influenced by the cancer's stage. It was discovered that MALAT1 expression was higher in stages I and II, decreased in stages III, and again rose in stage IV. Furthermore, the study samples from various populations may have an impact on the outcome. Results obtained from the GEO database for MALAT1 were also found to be consistent with that of the GEPIA database. It was observed that the level of MALAT1 varies in tumor tissue compared to normal tissue. Therefore, the expression level of MALAT1 can be said to be inconclusive throughout cancer stages and varies in different types of cancer as well as different sites of metastasis.

On the contrary, expression levels of AFAP1‐AS1 were found to be statistically significantly higher in tumor tissue compared to normal tissue according to both GEPIA and GEO databases. A high level of AFAP1‐AS1 was also correlated with a decline in both OS and DFS. Additionally, AFAP1‐AS1 expression tends to increase toward the later stage, specifically stage IV of cancer development, which often has signs of various types of invasions and metastasis [51, 52]. Therefore, it reinforced the outcome found in this meta‐analysis. In the case of HOTAIR studies, no concluding statement can be made, as the outcome from neither GEPIA nor GEO database was statistically significant. This may be explained by the fact that the sample size for HOTAIR studies was quite small.

Although the expression levels of MALAT1, HOTAIR, and AFAP1‐AS1 showed a statistically significant correlation with LNM in lung cancer patients according to the forest plots, the quality assessment tests demonstrated that the outcome for AFAP1‐AS1 is more reliable. Additionally, this outcome falls in line with that of the analysis with GEPIA and GEO databases for AFAP1‐AS1. However, the expression level of MALAT1 was found to be inconclusive in tumor tissue compared to normal tissue according to GEPIA and GEO databases. Therefore, AFAP1‐AS1 can be considered the most suitable biomarker for assessing the risk of LNM development from lung cancer compared to the other two lncRNAs.

Prognosis is important in oncology, as it can accurately predict the outcomes of treatment. It enables the implementation of clinical observations in a way that effectively considers the distinctive characteristics of both the patient and the tumor while making treatment decisions. This requires characterizing patients in terms of genetic and epigenetic properties that may influence the course of the cancer progression. This information can be used to predict the probability of developing metastasis, which can eventually be utilized for efficient planning and treatment strategies.

Several aspects of this study can be optimized in future studies. The literature mostly focused on the situation in East Asia because it included enrolled patients from China, Japan, and Taiwan. Should further pertinent literature from other fields be discovered, the study's findings might be more widely applicable. High values of heterogeneity can cause difficulties in interpreting the results. The potential source of heterogeneity for this study might be the following factors: (i) tumor specimen preservation methods were different after surgical resection; in this paper, samples were stored in liquid nitrogen until use, (ii) the studies included for analysis had different qRT‐PCR conditions and reaction systems; the difference in this literature might affect the results, (iii) the selected studies had slightly different standard parameters for grading the lncRNA expression profile into high and low, that is, the ideal threshold value had not been established, which could result in heterogeneity. Additionally, various factors including demographic parameters might affect the expression levels of lncRNA and, consequently, the outcome of the study.

In order to implement the outcome of this study clinically or to use it to predict LNM, it should be highlighted that this meta‐analysis investigates the expression pattern of lncRNAs in patients who have been diagnosed with LNM. It is based on observational studies, which are usually used to establish association and correlation, not causal relationships. Although the GEPIA database's stage plots demonstrate how lncRNA expression changes as cancer progresses, more research with a larger sample size and details on the dynamics of lncRNA expression as cancer develops toward metastasis is needed to determine which expression level is concerning at what stage of the disease. As lncRNAs’ role in cancer has been a field of great research interest in recent years, many lncRNAs have been discovered associating their role in various aspects of cancers, including their role as biomarkers, in prognosis, in metastasis, and in therapeutics. But among the pools of lncRNAs, there might be some that can be associated more strongly with a specific aspect of cancer. This analysis showed that among the three lncRNAs associated with metastasis of lung cancer, high‐level expression of AFAP1‐AS1 is more prevalent in lung cancer patients with LNM. Further research on this topic can increase the strength of the result of this meta‐analysis. Therefore, this analysis increases the utility of further research on AFAP1‐AS1 and its role as a biomarker of lung cancer metastasis as well as its targeting in therapeutics. Finally, it can also provide useful information to researchers, policymakers, and clinicians involved.

## Author Contributions

**Anha Tasnim:** conceptualization, investigation, writing – original draft, methodology, validation, visualization, writing – review and editing, data curation. **Afra Anjum Sumaiya:** conceptualization, investigation, writing – original draft, methodology, validation, software, formal analysis, data curation, writing – review and editing. **Abdullah Al Noman:** software, formal analysis. **Anika Tahsin:** software, formal analysis, writing – review and editing, visualization. **Abdullah Al Saba:** conceptualization, investigation, software, formal analysis, validation, supervision, writing – original draft, methodology. **Rubaiat Ahmed:** conceptualization, software, methodology. **Tahirah Yasmin:** investigation, supervision, resources. **A. H. M. Nurun Nabi:** project administration, conceptualization, supervision, resources.

## Conflicts of Interest

The authors declare no conflicts of interest.

## Supporting information


**Data S1** Questionnaire for Newcastle—Ottawa Quality Assessment Scale (adapted for case–control studies).

## Data Availability

The data that support the findings of this study are available from the corresponding author upon reasonable request.
